# Investigating the Impact of Isolation During COVID-19 on Family Functioning – An Australian Snapshot

**DOI:** 10.3389/fpsyg.2021.722161

**Published:** 2021-12-07

**Authors:** Jade Sheen, Anna Aridas, Phillip Tchernegovski, Amanda Dudley, Jane McGillivray, Andrea Reupert

**Affiliations:** ^1^Faculty of Health, School of Psychology, Deakin University, Geelong, VIC, Australia; ^2^Faculty of Education, Monash University, Clayton, VIC, Australia

**Keywords:** family functioning, family relationships, parenting, COVID-19, isolation, Australia, qualitative study

## Abstract

This study explored possible changes in family functioning from the perspective of parents during the COVID-19 pandemic. Thirty-four parents/guardians of children under 18 years completed a semi-structured interview, average length 47 min. Interviews focussed on changes in different aspects of family functioning including family roles, routines, and rules; parenting practices; communication and relationships; and strengths, challenges, and tensions. Data were analysed using reflexive thematic analysis applied in an idiographic and inductive manner to reduce the loss of individual experiences and perspectives. Four superordinate themes were identified: shifting family roles and boundaries throughout the pandemic; impacts on routines and relationships; opportunities and resourcing; and, experiences of support and unity. Gender differences were evident across some themes, particularly changing roles, workload and work-home boundaries. Challenges and tensions were frequently highlighted, particularly by “vulnerable” family groups such as those with children with disabilities. Parents also described a renewed sense of family and community that underpinned adaptive coping responses. The results highlight the importance of family connectedness in times of need.

## Introduction

The COVID-19 pandemic has had an unprecedented impact on the daily lives of families globally. To date, over 240 million people worldwide have been diagnosed with COVID-19 and over 4.89 million deaths have been reported.^1^

The World Health Organization suggests a Mobilise-Control-Suppress-Reduce-Develop approach to the systemic management of COVID-19 [World Health Organization [WHO], 2020]. Accordingly, various health mandates have been issued across countries in an attempt to slow the spread of the virus. The focus of this study is on the impacts of the Suppress aspect of the plan, including measures such as isolation, quarantine, and regional lockdowns.

The Australian Government Department of Health (2020a) defines isolation as the process of separating people with COVID-19 or suspected of having COVID-19, from those who are not ill. By contrast, quarantine separates and restricts the movement of individuals exposed to COVID-19 to ascertain if they become ill. The term lockdown has notionally been used to describe a series of government mandated restrictions on the movements of most community members, in an effort to reduce the spread of COVID-19, typically occurring when the spread of the disease becomes so widespread that more precise interventions are rendered less effective. During lockdowns, community members are separated from those outside of their household. This study focuses on the impacts of lockdown on family functioning. As this study details results from an Australian sample, Australian specific COVID-19 policies and guidelines will be considered. Nonetheless, implications for families in other countries will be highlighted.

**FIGURE 1:**
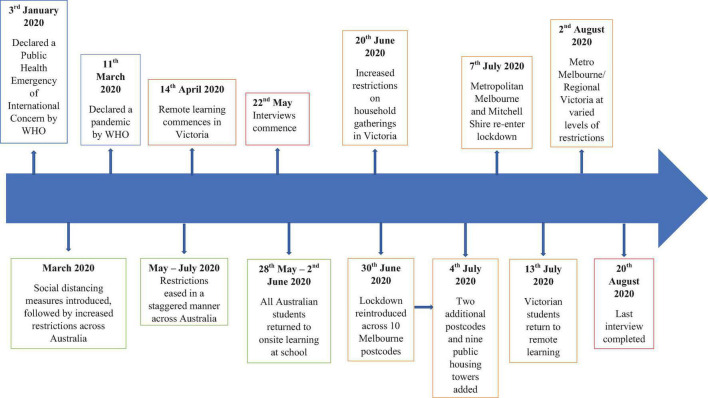
Key milestones in Australia’s response to COVID-19 and Victorian specific restrictions.

Since Australia’s first reported case in March 2020, strict social distancing and isolation guidelines have been employed to manage the progression of COVID-19. International borders were quickly closed and mandatory quarantine was introduced for returned travellers. Additionally, strict stay-at-home restrictions were implemented from late March to early June. Mandates across the country included social distancing and the use of protective equipment such as hand sanitiser and masks. Figure 1 provides an overview of key milestones in Australia’s response to the COVID-19 pandemic, providing context for the presented data.

Over the course of the pandemic, there have been local variations due to different state and territory governments jurisdictions (Australian Government Department of Health, 2020b). Each state and territory government has its own management plan, resources, and risks to navigate. For example, Victoria endured one of the longest and strictest lockdowns in the world following a second wave outbreak of COVID-19 in 2020. Victorian specific data has been added to Figure 1 to reflect the unusually long and harsh restrictions imposed.

### Impacts of Lockdown During the Pandemic

The absence of routine and social isolation associated with lockdown has had significant impacts on the physical and mental health of children (Chen et al., 2020; Singh et al., 2020; Wang et al., 2020) and adults alike (Fiordelli et al., 2020; Jurblum et al., 2020; Stamu et al., 2020; Ogden, 2021). For children, lockdowns are typically associated with prolonged school closures and increased confinement (Wang et al., 2020). When children are out of school, they engage in less physical activity and more screen use, report irregular sleep patterns and typically have less favourable diets (Brazendale et al., 2017; Wang et al., 2019). Studies examining the impact of lockdown during the COVID-19 pandemic also suggest an increase in behavioural concerns and poor sleep amongst children (Jiao et al., 2020) as well as increased irritability, inattention, and clingy behaviour across all age groups (Viner et al., 2020). Significant concerns have also been raised for “at risk” youth when schools, legal centres, and preventative services are not functioning fully, as they may have fewer opportunities to report harm or abuse safely (Singh et al., 2020).

Lockdowns also present significant challenges for adults. For example, a study of the impacts of stay-at-home orders on the mental health of adults from the United States (US) during the COVID-19 pandemic, found that lockdown led to significant reductions in measures of mental health (Adams-Prassl et al., 2020). Similar declines have been observed in the mental health of adult populations experiencing lockdown across the world, for example in Germany (Ahrens et al., 2021), Italy (Fiordelli et al., 2020), Greece (Fountoulakis et al., 2021), Canada (Gadermann et al., 2021), and the United Kingdom (UK) (Ogden, 2021). Lockdown has also been associated with an increase in family violence (Campbell, 2020; Gadermann et al., 2021), economic hardship (Fegert et al., 2020); increased alcohol consumption (Gadermann et al., 2021); a decrease in exercise (Deschasaux-Tanguy et al., 2021); and a global sense of loss and grief (de Jong et al., 2020). These findings are consistent with studies conducted during the SARS pandemic (Blendon et al., 2004) and natural disasters (Cao et al., 2013; Pujadas Botey and Kulig, 2014; Kotozaki et al., 2021; O’Donohue et al., 2021), suggesting a relationship between social isolation and poor social, mental, and physical health outcomes.

Overall, these results suggest that interventions such as lockdown are likely to impact the mental and physical health and well-being of children and adults. Perhaps for this reason, lockdowns have become a controversial topic (Schippers, 2020), with some authors suggesting that the impacts of lockdown on public health are far more harmful than COVID-19 (Joffe, 2021). Townsend (2020) adds that children will bear the brunt of the economic fallout from lockdowns, while also being the most damaged by it. While lockdowns continue to play a role in public health policy, more must be done to understand the impacts on families and communities.

### Coping in the Face of Crisis

In an attempt to minimise the deleterious effects of social distance and lockdown on mental health and well-being, researchers have turned their attention to potential interventions and protective factors. At an individual level, interventions such as life-crafting have been discussed. In general, having a clear meaning and purpose in life appears to facilitate good mental health and well-being. In lockdown, when activity is restricted and loss of purpose, employment and relationships abound, life-crafting may enable individuals to refocus and regain a sense of purpose and significance (de Jong et al., 2020). Life-crafting interventions help people find meaning in life by actively reflecting on their present and future lives, identifying specific goals, making concrete plans to achieve those goals and managing potential obstacles (Schippers and Ziegler, 2019; de Jong et al., 2020). A number of other interventions have also been explored as potential supports during lockdown including Acceptance Commitment Therapy (Landi et al., 2020) and a virtual reality-based self-help intervention (Riva et al., 2021).

With regards to protective factors, a number of studies have highlighted the importance of coping styles and social support, particularly from family members. Positive thinking (Budimir et al., 2021; Fuller and Huseth-Zosel, 2021), active stress coping (Budimir et al., 2021; Park et al., 2021), task-oriented coping (Orgiles et al., 2020), social support (Budimir et al., 2021; Park et al., 2021; Preston and Rew, 2021), and perceived family support (Huston, 2000; Mariani et al., 2020; Salin et al., 2020; Preston and Rew, 2021) have all been identified as protective factors for children and adults during the pandemic. Conversely, emotional coping styles in children (Orgiles et al., 2020), avoidant coping styles in parents and adolescents (Lorenzo et al., 2021), emotional and avoidant coping styles in adults (Mariani et al., 2020; Fluharty et al., 2021), and low perceived family and social support (Mariani et al., 2020; Rahman et al., 2020; Budimir et al., 2021; Gabarrell-Pascuet et al., 2021) have been correlated with greater anxious and depressive symptoms.

Collectively, these studies suggest that the impacts of lockdown and the factors that protect our mental health during lockdown, should be considered beyond an individual level, to at least include the relational. This paper will focus exclusively on the role of family as a protective factor in managing the impacts of lockdown.

### The Vital Role of Family

Healthy family functioning has the power to help or hinder individuals attempts to cope with major life events, such as a pandemic (Power et al., 2015). Cohesive and supportive family processes have been found to protect individuals from the negative effects of life stress (Hobfoll and Spielberger, 1992) *and* generate a variety of positive outcomes for children and their carers (e.g., Conger and Conger, 2004; Joel Wong et al., 2012). Through the course of previous pandemics, small factors such as the way in which parents communicated to their children about the event impacted their child’s subsequent feelings of fear and anxiety (Berger et al., 2021).

Cluver et al. (2020) suggest that the current crisis may actually elicit opportunities for strengthening family relationships. For example, stay-at-home orders are likely to result in more fathers working from home and spending more time with their children. Staying at home may also afford parents unique opportunities for flexible family routines in ways that would otherwise not be available to them (Cito et al., 2020). Early COVID-19 research (Evans et al., 2020; Gunther-Bel et al., 2020) support the assertion that these benefits may be present alongside the negative impacts of lockdowns on families. For example, Gunther-Bel et al. (2020) investigated the relational well-being of 407 Spanish adults 3 weeks into their first COVID-related lockdown. In response to an open-ended question in their survey, they found both relational improvements and deterioration. The benefits of increased connection were cited most frequently, followed by increased conflict. Similarly, Evans et al. (2020) surveyed 2130 families during the early stages of lockdown in Australia. Predominant themes identified in their open-ended survey question centred on loss and challenge, with many families reporting mental health difficulties and strained family relationships. This experience was not universal, however, with some families reporting positive benefits and meaning including opportunities for more time together along with appreciation, gratitude, and tolerance. The polarised nature of these findings is consistent with studies from the broader literature following family functioning after a period of trauma such as parental mental illness (Marston et al., 2016); in the wake of widespread socioeconomic stress (Margerison-Zilko et al., 2016); and, following natural disasters such as earthquakes (Cao et al., 2013) and fire (Pujadas Botey and Kulig, 2014). Broadly, these studies suggest that healthy family functioning can be an important protective factor, but also presents as a risk when conflict and poor mental health dominate.

Although these early studies provide important insight into family relationships, further qualitative research is required to elaborate on our understanding of key aspects of family functioning such as routines, rituals, and rules, during the pandemic (Prime et al., 2020). Such research will allow us to better understand how families function during periods of severe and unexpected stress, potentially informing future trauma interventions and responses. Further, exploring the strengths and coping strategies unique to family units during this time of disruption may provide insights and direction for future interventions and supports, both during the pandemic and beyond.

The overall aim of this study was to explore Australian parents’ perspectives of possible changes to family functioning during the pandemic and associated lockdowns. Specifically, this paper aimed to:

1. Investigate the impacts of the COVID-19 pandemic and lockdown on family functioning including family roles, routines, and dynamics;
2. Investigate what, if any, changes have occurred in family relationships including the ways in which family members interact, communicate, and support each other;
3. Explore any challenges and tensions that have been experienced in family life as a result of the pandemic and lockdown; and,
4. Explore the strengths and coping strategies that families are utilising during the pandemic and lockdown.

## Materials and Methods

### Participants and Recruitment Procedure

Thirty-four parents/guardians of children under 18 years were recruited from Australia using advertisements on online forums (e.g., social media platforms, Facebook, Instagram, LinkedIn). A snowball method was employed, whereby participants were invited to share the information with others who may be interested in participation. The mean age of participants was 39 years, with ages ranging between 24 and 60. All participants had experienced at least one period of lockdown and the resulting social isolation prior to interview; however, none had been formally isolated or quarantined due to contact with COVID-19. See Table 1 for further demographic details.

**TABLE 1:** Demographic details.

	*n*	%
**Gender**		
Female	30	88
Male	4	12
**Age range**		
Under 30	1	3
30–34	14	41
35–39	1	3
40–44	11	32
45–49	3	9
50–54	2	6
55–59	1	3
60 and over	1	3
**Relationship status**		
Married/*de facto*	32	94
Single/divorced	2	6
**Ethnicity**		
Anglo-Saxon	25	73
Singaporean	2	6
Chinese	1	3
Sri Lankan	1	3
Colombian	1	3
Vietnamese	1	3
Aboriginal	1	3
Hong-Kong	1	3
Spanish	1	3
**State**		
VIC	32	94
NSW	1	3
SA	1	3
**Region**		
Urban	28	82
Regional	3	9
Semi-rural	1	3
Rural	2	6

### Procedure

Ethics approval for this study was provided by the Deakin University, Human Ethics Advisory Group Health (HEAG-H 70-2020). Consenting participants completed a semi-structured interview ranging from 21 to 79 min in length, with an average of 47 min. Interview questions were developed by the researchers and informed by previous literature on the impacts of disasters on family functioning and the McMaster Family Assessment Device (FAD; Epstein et al., 1983). The FAD, based on the McMaster Model of Family Functioning, considers aspects of family functioning such as problem solving, roles, communication, affective responsiveness, affective involvement, and behaviour control (Epstein et al., 1983). The FAD provides a useful theoretical conceptualisation of family functioning that might be used to inform family supports and tailored interventions.

The interview schedule included questions about the key areas of family functioning including family roles, routines, and rules; parenting practices; communication and relationships; and, strengths, challenges, and tensions. When responding, participants were asked to reflect on changes “since the beginning of the COVID-19 pandemic.” Participants were also asked to reflect specifically on changes observed during lockdown. Most participants resided in Victoria and were under lockdown when the interviews were conducted between May and August, 2020. Those who completed interviews when restrictions were lifted or who lived in other states were asked to specifically reflect on their last period of lockdown when responding to these items.

Due to COVID-19 restrictions, interviews were completed *via* the Zoom video conferencing app. With participants’ permission, interviews were video recorded and transcribed with identifying details omitted. Once complete, transcriptions were checked against the original recording for accuracy before being sent to participants for member checks. Participants were given 2 weeks to provide feedback on their transcript before the data were included in the group analysis. Only one participant stated *via* email response that they wanted to clarify a comment made during interview, no further changes were requested.

### Data Analysis

A reflexive thematic analysis, described by Braun et al. (2019) was employed. Within this approach, themes are conceptualised as meaning based patterns, evident in both explicit and conceptual ways. As lived experience of an unusual event was central to this study, the thematic analysis was completed in an idiographic manner to reduce the loss of individual experiences and perspectives. Although the interview questions were informed partially by the FAD, the analysis was inductive and data driven.

Authors JS, AA, PT, AD, and AR were randomly allocated 6–7 transcripts each. The authors read and undertook a full analysis on each transcript. This involved reading and then re-reading each transcript and highlighting quotes which held meaning for the participant, the researcher, and the research question. Transcripts were then coded to identify verbal content at a micro level. Codes were clustered to identify key concepts within the transcript. Comparisons and contrasts of these concepts were then made across transcripts through recursive and reflective discussion. Reoccurring concepts were identified and developed into provisional super- and sub-ordinate themes. Authors then reviewed their allocated transcripts against the themes, highlighting relevant quotes as well as contrary cases. This stage of analysis also served to check for any relevant data that had not been captured by the themes. During a second collaborative discussion, the themes were revised and finalised. Themes were also considered in relation to participants’ demographics. The final themes are listed in Table 2.

**TABLE 2:** The impacts of COVID-19 on family functioning: superordinate and subordinate themes.

Superordinate themes	Subordinate themes
Shifting family roles and boundaries	It’s all a juggle: balancing roles
	Re-navigation of work-life boundaries
	Discipline
Routines and relationships	Rituals of connection
	Time together, but no time apart
	Communication shifts
The haves and the have nots (opportunities and resourcing)	Physical resourcing
Support and unity	Support lost but also gained
	Learning more about each other’s world

In creating thematic narratives, the authors tabulated the themes and for each participant, reflected on the presence of related data or conversely, refuting data to ensure that individual perspectives were not lost. The lead author then drew from this resource when writing, double checking back to original transcripts to retain authenticity.

### Reflexivity

The first author led the data collection and analysis. At the time of this study, she was a Clinical Psychologist, Associate Professor and parent living in Victoria, Australia. Her interest and perspectives on the subject matter were informed by a lived experience of parenting during the COVID-19 pandemic and a history of working with families as a clinician and researcher. The research team brought expertise in research methods, including qualitative research, and experience from research and psychological practice involving children and families living with parental mental illness. Although these experiences shape the world view of the research team, these have been acknowledged and several steps were taken within this project to maintain the rigour of the findings including a reflexive journal, engaging in self-reflection and robust, regular discussion within the team.

## Results

During analysis, four superordinate and nine subordinate themes were identified within the data that related to the research questions presented in this study. The themes are identified in Table 2. Quotes are cited with gender/age/relationship status to provide context. State was provided if not Victoria.

### Shifting Family Roles and Boundaries

Participants reflected on the shifting nature of family roles and boundaries brought about by lockdowns during which schools closed and parents not in frontline roles were required to work from home. Subthemes related to the impacts of juggling multiple family roles; re-navigation of work-life boundaries; and boundaries around discipline and parenting.

#### It’s All a Juggle: Balancing Roles

Participants reflected on changes in their workload and the need to balance many roles throughout the course of the pandemic. “Dramatic shifts occurred for parents of school aged children during periods of home schooling; *it’s like I feel like the pressure of keeping up with the home duties, the cooking and the cleaning, the working, and now the home schooling again, it kind of is a little bit too much*” (F, 44, *de facto*) and “*it wasn’t ideal to be teaching boys while we were working*” (F, 42, married).

The strain of balancing these multiple roles was exacerbated during lockdowns due to reduced outside supports such as childcare and access to extended family members. Female participants described absorbing much of this additional workload. In the words of one participant, “*I feel like I’m doing 95% of the mundane work because that’s just expected of me*… *the patriarchy is rife*” (F, 40, married). Likewise, participant 5 (F, 30, married) said, “*he’s [husband] not too bad in terms of helping but there are certain things it doesn’t cross his mind to help with*.”

In male participants not involved in primary caregiving, there was often a sense of intrusion to the day to day running of the household when they were suddenly required to work from home, “*it suddenly disrupted what was a pretty clear way my wife and my kids got along. And then suddenly I’m involved*” (M, 54, married).

In some families, parents asked children to assume home duties, e.g.,

…*they’ve been doing a lot more cooking with me, a lot more part of the household chores* (F, 40, married).

*I used to do all the housework because basically*… *But since the lockdown I can delegate some of the housework to my kids* (F, 43, married).

#### Re-navigation of Work-Life Boundaries

Participants described the need to re-navigate usual work-life boundaries during lockdowns. For some, this was challenging with participants reporting an increase in time allocated to paid work, even as the demands of travel and other workplace activities decreased.

…*the boundaries of normal have shifted, you know. And it’s different if it was just a day, but we had my son at home for six weeks and as delightful as it was, the new normal became, someone’s doing work late, and I was up at three o’clock one morning trying to get work done* (F, 42, *de facto*).

This was particularly challenging for the male participants used to working outside of the home.

*I’d go from my desk at work, to helping kids in schooling, because they found it – in some cases they found it really difficult, because they couldn’t put their hand, or speak to the teacher. It was quite daunting* (M, 54, married).

*A lot more interruptions to the work concentration. A lot more shorter, sharper periods of focus, periods of work* (M, 55, married).

“Both male and female participants described the omnipresent nature of work when at home; *it’s been hard to shut off that’s for sure. The computer’s there and it’s on*” (F, 46, married).

Similarly, parents had to negotiate how they shared the home space so that various family members could continue with their regular activities.

…*she [child] just does a lot of gymnastics and dancing and things so she needed to use the majority of the space in the living and dining room. We didn’t really want to be walking in and out of the video kind of thing so I just found it was a bit restricted on what we could do as a family while she was doing that all afternoon* (F, 40, married, NSW).

Changes to work and home were ubiquitous and multilayered:

…*there’s the personal layers, there’s the COVID layers, there’s the son layers, the work layers and all that on top of each other it’s just*…*I mean if you’re talking about things that change at home, it’s changed everywhere and it hasn’t always been bad but it’s been challenging trying to manage everything simultaneously* (F, 42, *de facto*).

#### Discipline

Participants described changes in relation to discipline with some feeling that being home made it easier to follow through.

*I think because we’ve been home more and around more we’re making sure that he actually follows through with his chores like unstacking the dishwasher and all that sort of thing. Whereas before if we were busy it was just like, gosh, I’m just going to do it, you know, he’s engaged in something else* (F, 50, *de facto*).

Conversely, others described being more lenient.

*I probably have been a bit more lenient, in that I’m like, well we’re all stuck here so there’s no point, I don’t know, I’ve probably let them get away with a bit more, you know climbing up the walls a bit* (F, 30, married).

Despite expressed preferences to limit their children’s screen time, many describing allowing children to use electronic devices as participant 31 (F, 50, *de facto*) explained, “*We’ve sort of thrown out a lot of boundaries that we had. Boundaries around technology use, my goodness, he used to get like, you know – he was a real non-technology kid*…*He’s doing it everyday now*.”

### Routine and Relationships

Periods of lockdown impacted on the daily routine of most families, with associated relational shifts observed. Most notably, lockdown impacted on family members rituals and routines of connection and their opportunity for time together, though this came at the expense of time alone. Frequent communication within the parental unit appeared central to navigating these changes.

#### Rituals/Routines of Connection

For all participants the various rituals and routines they had to connect with others changed, but in different ways across the sample. Some described losing these opportunities during lockdown; “*we (the parents) used to go and get a coffee every Thursday together and that would finish off our week, and obviously with COVID you can’t do those sorts of things*” (F, 34, married).

As lockdown progressed, many described new family routines. This was sometimes purposeful as participant 9 (F, 34, married) explained, “*We try to make sure we get every day – every night and go for a walk or something together and that’s probably a new inclusion because we have dropped all the other stuff off*.” Others also described new family routines that evolved around physical activities, “*We were doing a lot more – you know, riding our bikes, going for walks, going to local parks every time we could, taking our breaks throughout the day* (F, 42, married).” These new routines prompted participant 4 (F, 40, married) to reflect on how her family used to spend their time:

… *now you sort of look back on it and go is that stuff actually necessary or is it better that we have this family time which I think has been really rich and beautiful rather than fulfilling these expectations that we may put on ourselves about what we need to be achieving so I don’t want to go back to those old ways of living*.

Spending more time as a family allowed participants to reflect on a “*simpler way of living*” (F, 40, married) which in turn “*strengthened us*” as a family unit (F, 33, married). Participant 9 (F, 34, married) described this opportunity as being able to “*enjoy some of the little things that we might not have stopped to think about or realise that we were experiencing*.”

Connecting with extended family also changed during lockdown, as noted by participant 16 (F, 33, married) “*we have this real separation from our (extended) family at the moment and we’re relying on totally online face-to-face kind of methods of staying in touch*” though some reflected on the benefits of the reduction in social commitments and obligations afforded by lockdown.

…*though of course we love our extended family and want to see them it’s been really nice to have that breather as well and just time for our family to just go for walks in the park and everything’s slowed down a little bit* (F, 32, married).

#### Time Together, but No Time Apart

All participants described spending more time together as a family due to interrupted activities outside of the home. Participant 1 (F, 33, married) indicated that her husband, “*hasn’t been able to golf on weekends so it’s just been us in the home and he’s loved it because he’s had really quality time with our son and me*.” Participants recognised the benefits of this increased family time for the couple, “*spending more time together I think for us has been good*” and for the family unit:

*I guess a healing time almost in a way because we feel like we’re eating better, we’re sleeping better, we’re spending more time* together (F, 50, *de facto*).

*I definitely think there’s been a really lovely – yeah, just a closeness I think that comes from it* (F, 37, married).

Participant 7 (F, 40, married) reflected that, “*It’s monotonous and it’s challenging but really simple things like we have dinner at the table every night together. The contact time that my husband has had with the kids is a lot more*… *it’s been really good*.”

Conversely, the increased time together resulted in tension and/or conflict for some couples and families with participant 13 (F, 44, married) observing “*maybe less tolerance*.”

*I’m probably more short with him you know I haven’t had that, like again on days where I’m not working and I’m just home and I’m probably at my wits end I might get a bit short with him* (F, 33, married).

*(The family were) always in each other pockets. So, it created an element of tension at times* (M, 54, married).

Some participants commented that the gains in family time came at the expense of “*less me time*” (F, 32, married) and where it has “*been quite difficult to not have any of my own time you know like the kids are with me 24/7*” (F, 40, married).

#### Communication Shifts

When reflecting on the ways in which families managed changes in routine and connection during lockdowns, the importance of communication was observed both explicitly and implicitly. Some participants described working more as a team with the other parent:

*I would just really had to go what’s your day looking like tomorrow? Who’s stopping to, you know, to do – cook lunch and have things ready? And when are we doing this and when are we squeezing in time to do these activities? So I guess, yeah, in that sense, it was probably more the team planning around what would happen each day, that I saw were the main difference* (F, 42, married).

Participants that reflected on strong and open communication within the parental unit, typically reported more positive affect during lockdown than those where communication was poorer.

*We certainly went through a period of being very, very cranky with each other and having much poorer communication than would be desired. We were definitely getting a bit cranky for a bit there*… (M, 34, married).

### The Haves and the Have Nots (Opportunities and Resourcing)

For some participants, physical resourcing and space was an issue, where “… *everyone was kind of on top of each other in the day*” (F, 24, married, SA). Others were more fortunate because of access to space including backyards and parks. Likewise, others were in financially stable positions and on that basis “*talking to the boys about how privileged we*” (F, 42, married). Conversely, job insecurity brought its own stress:

*He just had to hope and pray that he wasn’t going to lose his job and he had to speak to his manager to try and work out how long the pay cut was going to be and things were going to go back to normal. He was just stressed and down in the dumps for a bit* (F, 34, married).

Although some participants found that they had more resources during the pandemic “*it was more beneficial financially for us because we didn’t have to pay for care*” (F, 34, married), those impacted by job insecurity noted pressures, “*there were redundancies made at his company pretty early on, and so he’s being cautious and conscious, and so, he’s just trying to do, like the absolute best he can and do as much as he can so that he can show like, hey, I’m really needed here*” (F, 33, married).

### Support and Unity

Participants reflected on the importance of support in managing the stressors associated with the pandemic in general and lockdown specifically. The loss of support experienced by many participations was therefore challenging, though examples of accommodation and adaptation were also provided.

#### Support Lost and Gained

A loss of support was identified during the pandemic by some participants. The loss included formal and professional support with medical appointments cancelled early on or pivoted to online support such as children’s psychiatric appointments (F, 60, divorced) or church services (F, 33, married). Some reported a loss of support from extended family and where

…*my parents in particular are really big on taking our daughter for a night over the weekend, so we would often get you know maybe one or two weekends a month, or night you know a week where she would be with parents and we could just have some decompression time* (F, 33, married).

These losses were most often observed in families with existing vulnerabilities, such as those with children with developmental disabilities or new parents. Participant 10 (F, 33, married) noted “*I now feel like things are being taken from me*… *just silly things like having to cancel a baby afternoon tea thing*… *and then post birth the anxiety and stress about having, not having parents around to basically help you*.”

Others described a strengthening of existing family relationships.

*Our boys*… *it’s forged their relationships with each other because they’ve only had each other as options* (F, 40, married).

*I think we always knew we [parents] were a good team but going through something like this and trying to manage our unit I think has given us pretty good confidence to tackle anything else that comes our way* (F, 34, married).

This strengthening of existing family relationships was developed in the face of “*really fun times*… *really boring times and there’s been frustrations but I think it has made us a bit tighter*” (F, 40, married).

#### Learning More About Each Other’s World

Lockdown gave families an opportunity to learn more about each other. Some described their children appreciating more about their work:

…*they (the children) could kind of maybe could see more, it was tangible then, they could see my husband was, he’s a teacher, so they could see him remote teaching in and they could see I was busy, my groups supervision and there was 6 students on the screen or something, they’re like oh Mum actually does do stuff, like she doesn’t just turn up to an office and magic happens* (F, 45, married).

Some described learning more about their children’s learning:

*I did enjoy how much I got to see how they learn and what motivates them at school and what topics they choose when they’re giving flexibility and freedom to make choices* (F, 42, married).

Participant 29 (F, 46, married) sums up this theme by stating, “*I think because we understand better what each other’s days like now*.”

## Discussion

The findings from this study provide insight into the experiences of Australian families during the COVID-19 pandemic and the impacts of lockdown. According to those interviewed, parents experienced changes in daily family life, including challenges juggling various commitments and separating work from home life. For some, family bonds strengthened and they learnt more about each other’s worlds as they spent more time together. For parents, this came at an expense of “me time” with some couples and sibling units also experiencing additional conflict. Changes in workloads, chores, screen time allowances, and discipline were observed. The pandemic changed the ways in which they connected with extended family and community and highlighted the need for more frequent communication within family units. There were also particular gendered experiences in relation to housework and child caring arrangements.

### Family Life

Parents noted that a renavigation of family roles was required to manage new demands of working and schooling from home. In some instances, children took on greater responsibilities. Although some participants described the additional chores, e.g., extra cleaning associated with being at home more, some reflected that their children simply “had more time” due to the cessation of most extracurricular activities during lockdowns. These findings are consistent with other research highlighting the significant changes that have occurred for families throughout the pandemic, from shifts in roles and routines (Fegert et al., 2020) to the allocation of chores (Evans et al., 2020). Of concern, shifts in daily routine and working conditions have been found to negatively affect parents’ psychological well-being, also exposing children to a significant risk for their well-being (Cusinato et al., 2020).

Positively, parents in this study noted that there was more time to engage as a family during lockdowns due to a decline in social commitments, providing in some instances a “relief” for busy parents. Similarly, Fegert et al. (2020) argued that the absence of appointments, guests, and travel as a consequence of the pandemic, could bring rest and relaxation into family life. The notion that these changes could be a “relief” calls into question the busy nature of modern family life and responsibilities and obligations attached. Perhaps families should be encouraged to reflect on the benefits of these shifts and in a similar manner to life crafting methodologies (de Jong et al., 2020), integrate learnings from the present into an ideal family future, setting goals that may allow for an improved family/life balance. Understanding the barriers that may exist for families wanting to retain positive change when lockdowns end is essential, highlighting the need for longitudinal research to investigate change over time.

The challenge of managing a healthy work/life balance was also highlighted for participants during lockdown, wherein for most, all activities of daily living occurred in the one (home) environment. In this sample, the outcomes were an increase in work hours as work became omnipresent and a need to juggle physical space to allow for home schooling, work and in some cases leisure activities in the one space, with parents having to find ways to negotiate the “the physical space in the house.” While few studies have examined the importance of physical space in the home during lockdown, a study of adults in Oslo, Norway, found an increased use of urban green space during lockdown, with a 291% increase in outdoor recreational activity reported (Venter et al., 2020), suggesting a change in the ways in which we use our environments. Further research is needed to explore this issue and the impacts on family functioning and well-being.

### Parenting

With more time together, parenting was a point of interest, as reflected through changes in family rules, boundaries and communication. The family stress model (Conger and Elder, 1994) suggests that when caregivers are faced with highly elevated levels of stress, such as may occur in a global pandemic, their mental and emotional resources are drained, challenging their capacity to provide positive leadership in their family. According to Prime et al. (2020), a consequence of this stress may be an overreliance on less effective parenting approaches such as harshness or authoritarian parenting. Consistent with this hypothesis, Brown et al. (2020) found that parents experienced cumulative stressors through COVID-19 which impacted on their mental health and increased child abuse potential. Likewise, Evans et al. (2020) found that the COVID-19 pandemic increased parents stress and decreased their opportunities for respite, while Feinberg et al. (2021) found large deteriorations in child internalising and externalising problems and parent depression in the early stages of the pandemic.

The current study contributes to the growing literature suggesting that lockdowns both supported and potentially challenged typical household rules. Some participants reported that more time at home enabled them to better communicate rule transgressions with their parenting partner and to follow up with consequences more thoughtfully and consistently than they would have otherwise. These relational level changes reflect the importance of communication and consistency when parenting. Other reports, sometimes by the same participant, noted that the lack of alone time, coupled with additional demands of home schooling and work, resulted in periods where they were more “lenient.” An implicit suggestion in the interviews was that parents relaxed boundaries at the time, due to the fatigue of being at home and managing increased responsibility. This is consistent with elements of the family stress model.

In all but one household, screen use was the most frequently relaxed boundary. Some parents cited the need to support screen time so that children could connect with extended family and friends during lockdown and a need to “give them some way to socialise.” Others noted that the combination of poor weather, limited space and “lockdown fatigue” contributed to the relaxation of screen rules. This finding was expected based on research conducted both prior to and during the COVID-19 pandemic, suggesting that children spend more time in front of screens when not physically attending school (e.g., Brazendale et al., 2017; Evans et al., 2020).

In some instances, participants noted that a relaxing of rules at home was not the result of increased pressure or less parenting capacity, as could easily be assumed. Instead, it was a thoughtful reflection of their child’s need for leniency during a stressful time. This was reflected in comments from parents noting that screen rules needed to be relaxed to allow their children to maintain important social contact or giving them additional warnings if they misbehaved to allow for expected distress. These examples appear more illustrative of family flexibility, a key component of family resilience (Walsh, 2003).

Interestingly, a shift toward more punitive parenting methods was not observed in this study. The more personal nature of qualitative interviews could potentially inhibit disclosures of punitive parenting approaches in some parents, suggesting the need for survey based investigations into the matter. While not directly investigated, based on comments made in the interviews participants also didn’t appear to have many of the risk factors associated with more punitive parenting, such as economic distress, food insecurity or high depressive symptoms (Brown et al., 2020; Rodriguez et al., 2021). These results highlight the importance of both design and sampling methods in research and suggest that the results need to be considered in the context of the broader literature to gather a more accurate, generalisable picture of parenting during the COVID-19 pandemic.

### Gender

Gender differences were observed in the expression of some themes in this sample, most notably in respect to work-home boundaries and time together. The majority of men in the sample worked from home during lockdowns, as few were in frontline roles. While both sexes reported an increase in workload associated with lockdown due to home schooling, increased housework and caregiving, more challenges were raised by male participants when navigating a work-home balance. Four of five men in the sample reported stress, at least initially, when they could not complete tasks uninterrupted. It is possible that men typically experience a clear delineation between each domain in their life, thus presenting a challenge when asked to switch roles quickly. By contrast, women who also worked from home noted an increase in workload, such as housework and childcare responsibilities, but rarely commented on the challenge of balancing work-home duties. It may well be that the female participants were practiced at balancing these roles, and accordingly, the difference during lockdown was less evident. Findings regarding perceived inequalities in the division of labour at home, are consistent with those of Hjaìlmsdoìttir and Bjarnadoìttir (2021) who reviewed the real time diary entries of 37 mothers in Iceland. The authors found that the division of tasks and household chores lay with women, who also assumed a greater mental load during the pandemic as they tried to keep everyone safe and calm at home. Gender differences in the division of household labour during the COVID-19 pandemic have also been observed in the UK (Xue and McMunn, 2021), US (Zamarro and Prados, 2021), and Australia (Craig and Churchill, 2021).

The men in this study also commented on the benefits of increased time with their children. Encouragingly, this is consistent with reports from Evans et al. (2020) regarding increased quality time spent between fathers and their children in the initial stages of the pandemic, and suggests this was ongoing, at least within the timeframe of the current study (May–August 2020). In Australia, women typically hold primary responsibility for child rearing following the birth of a child, with many moving down to part-time employment (Craig and Sawrikar, 2009). For many men, full time employment, coupled with long periods of time spent in transit, detract from family time. The benefits of more time in and the subsequent impacts on the family unit should be carefully considered by workplaces and policy makers. Although some workplace flexibility is offered in many roles, staff can be reluctant to take up the opportunity, particularly in a concerning economic climate, due to perceived pressure to show they are “really needed” by their employer. Encouraging use of flexible work arrangements and role modelling this at every level of an organisation is essential to ensure that the observed benefits of lockdown are not lost.

### Resilience

Resilience has become a point of interest for many researchers throughout this pandemic, given the direct and significant physical, psychological, and economic costs of COVID and associated lockdowns. Historically, it is rare for one trauma to have a collective impact. However, COVID-19 appears to have shattered the collective assumptions and worldview of many (Walsh, 2020), resulting in a resounding sense of grief and loss (de Jong et al., 2020). This was reflected in passing comments from participants, who noted that they had “taken for granted” the ability to leave their homes, visit interstate loved ones and shop for supplies without limits or restrictions in place. When lockdowns threatened these freedoms, participants were forced to identify new ways of coping and where possible, to reframe their distress. Comments related to the benefits of lockdown as family members learned more about each other’s worlds and reflected on “*a healing time*” may illustrate these mechanisms in action.

In a family context, resilience has been defined as the “capacities in family functioning to withstand and rebound from adversity” (Walsh, 2020, p. 904). This study sought to understand the impacts of the COVID-19 pandemic on family functioning from the perspective of parents and further, how they coped with the disruption of lockdown. The families in this study generally provided excellent examples of family resilience.

Parents in the study reflected on the loss of rituals of connection but also, ways in which this loss was accommodated and managed, with new rituals of connection developed. Games, walks and family meals became mainstays during lockdown, with participants reflecting on the importance of “the little things.” Open communication appeared to facilitate or at least accompany positive adaptation, with those families who reported a fairer distribution of labour and sense of “teamwork” typically reporting conscious, purposeful communication to manage change. These reports are consistent with those of Power et al. (2015), who investigated resilience in families where a parent has a mental illness. These authors noted that families developed resilience through processes such as regular family rituals and routines. They also noted that open communication, in this instance regarding mental illness, enabled families to cope when parents were unwell. Although the focus of the communication differed, there is clear consistency in the building blocks of family resilience in these two very different, but challenging, circumstances.

Finally, parents that reported greater distress and less resilience typically noted that lockdowns “worsened” existing vulnerabilities. For example, families with children with disabilities reported greater stress related to home-school and new parents reported greater stress with the loss of extended family and social support.

### Community

Walsh (2020, p. 903) notes that we are “relational beings” who mobilise kin and social support to cope. Lockdowns, and to a lesser extent social distancing, have resulted in significant distance between extended family members at a time when support is most needed, as social support assists in regulating emotions (Brock and Laifer, 2020). Outside of lockdown, many families are still in a state of self-imposed exile as they hold concerns over spreading the disease to a loved one.

The impact of this loss of broader support and community was reported by participants, particularly those in vulnerable circumstances. As noted by Prime et al. (2020), for children in vulnerable families, the loss of social supports outside of the home is a significant one. In families with fewer apparent vulnerabilities, such as two parent families, the loss of extended family support was in some ways offset by a greater sense of community in their local area. Although challenges were certainly noted, for many parents this seems to be weighted equally with the gains associated with more time together, learning more about each other’s worlds and strengthening community bonds. This is consistent with the findings of Gunther-Bel et al. (2020), who reported two overarching themes in their data: relational improvement and deterioration. This study also found, similarly, that the overall prevalence of improvement themes (61.7%) exceeded deterioration themes (41.0%), with increased (re)connection and conflict atmosphere cited most often.

Globally, the results appear to reflect changes that have impacted families at macroenvironmental, relational, and individual levels (Huston, 2000; Salin et al., 2020). At the macroenvironmental level, changes were observed in social and extended family support, managing work-life balance and managing multiple roles (particularly for parents’ home schooling). Relational changes observed in this study included time together as a family with an opportunity to learn more about each others’ worlds, the development of new rituals of connection, changes in communication, and changes in discipline when parenting. Positive change was supported when families were flexible and openly communicated. Individual level change includes shifts in personal time for parents.

## Future Directions

Walsh (2020, p. 904) asserts that healing and resilience in response to loss and other major disruptions does not mean that we quickly “bounce back.” Rather, she asserts that both are forged gradually over time. Hence, this study will continue longitudinally, following each family at regular intervals to understand their healing journey. Although future events such as periods of lockdown and rates of community infection cannot be controlled, following these families through possible changes should provide further insights into how family resilience is fostered but also, maintained or threatened. As outlined by Reupert (2021), there have been seven global health crises in the last 20 years; COVID-19 is not likely to be the last. It is critical that we gain further understanding about how families function during these times, from the perspective of children, and their parents.

Clinically, this study highlights the importance of open communication for families when navigating change. The study also highlights the importance of connectedness. Families in this study learnt more about each other’s worlds and developed new rituals and routines of connection with more time spent at home. Whilst participants expressed some challenges, they found ways to connect with extended family and community, either online or when exercising in their local area. Participants considered these strategies to be important protective factors and thus should be assessed by clinicians supporting vulnerable groups experiencing pandemic related periods of isolation.

## Limitations

It is acknowledged that the sampling techniques used in this study (i.e., snowball recruiting and advertisements), may have introduced bias toward middle class families with fewer vulnerabilities. For example, the sample skewed toward women, participants who reported no episodes of domestic violence and little economic distress, which is largely inconsistent with existing research that has highlighted an escalation in abuse, violence, and economic distress since the pandemic and lockdowns commenced (see for example, Bouillon-Minois et al., 2020; Fegert et al., 2020). Further, while robust for a qualitative study, the sample size of 34 participants does limit the generalisability of the results.

The sampling period is another limitation. Although there are advantages to undertaking the interviews between May 2020 and August 2020, the two most significant periods of lockdown in Victoria, the rapidly fluctuating nature of the Victorian lockdowns (e.g., length, severity of restriction, home schooling) means that participants may have reflected on different conditions when they completed their interviews.

## Conclusion

Since the commencement of the COVID-19 pandemic, the day to day lives of families have changed. Lockdowns have commonly been associated with stress and increased conflict as families negotiate changes in roles, attempt to find a healthier work-life balance and navigate the loss of extended family and social support. Vulnerabilities appear heightened at this time, suggesting the need for targetted support directed toward vulnerable groups and should lockdowns continue, the redevelopment of open community spaces in impoverished areas to support families to engage in positive coping strategies, such as exercise. Protectively, many families used the time together at home to connect and learn more about each other’s worlds. For some, there was a sense of relief associated with a quieter schedule and improved family connection that should be considered as we plan for the future. Many parents also described a renewed sense of family and community that underpinned adaptive coping responses. The results highlight the importance of family connectedness, flexibility, and communication in times of need.

## Data Availability Statement

The raw data supporting the conclusions of this article will be made available by the authors, without undue reservation.

## Ethics Statement

The studies involving human participants were reviewed and approved by the Deakin University, Human Ethics Advisory Group Health. The patients/participants provided their written informed consent to participate in this study.

## Author Contributions

JS, AA, AD, and AR were involved in the study design, execution, data analysis, and write up. AA conducted majority of the interviews. JS led the write up and wrote the first draft of the manuscript. PT was involved in data analysis and write up. JM was involved in write up. All authors contributed to the article and approved the submitted version.

## Conflict of Interest

The authors declare that the research was conducted in the absence of any commercial or financial relationships that could be construed as a potential conflict of interest.

## Publisher’s Note

All claims expressed in this article are solely those of the authors and do not necessarily represent those of their affiliated organizations, or those of the publisher, the editors and the reviewers. Any product that may be evaluated in this article, or claim that may be made by its manufacturer, is not guaranteed or endorsed by the publisher.

## Footnotes

1
https://www.who.int/emergencies/diseases/novel-coronavirus-2019
